# Renal tubular damage as an independent risk factor for all-cause and cardiovascular mortality in a community-based population: the Takahata study

**DOI:** 10.1007/s10157-024-02592-6

**Published:** 2024-11-14

**Authors:** Takaya Suzuki, Kazunobu Ichikawa, Natsuko Suzuki, Masafumi Watanabe, Tsuneo Konta

**Affiliations:** 1 Department of Cardiology, Pulmonology, and Nephrology, Yamagata University Faculty of Medicine, Yamagata, Japan; 2 Department of Public Health and Hygiene, Yamagata University Graduate School of Medical Science, 2-2-2, Iida-Nishi, Yamagata, 990-9585 Japan; 3 Institute for Promotion of Medical Science Research, Yamagata University Faculty of Medicine, Yamagata, Japan

**Keywords:** Albuminuria, Chronic kidney disease, General population, Renal tubular damage, Urinary beta 2-microglobulin

## Abstract

**Background:**

Renal tubular damage plays a crucial role in the development of end-stage kidney disease, a risk factor for cardiovascular events and mortality. However, the relationship between renal tubular damage and all-cause and cardiovascular mortality rates in the general population remains unclear. To address this gap, we conducted a cohort study in the general population using the urinary β2-microglobulin-creatinine ratio (UBCR) as a marker of renal tubular damage.

**Methods:**

This study included 3427 residents aged ≥ 40 years in Takahata, Japan. We examined the association between the UBCR values in single-spot urine samples at enrollment and all-cause and cardiovascular mortality rates within a median follow-up of 9.2 years.

**Results:**

The participants were divided into two groups based on their UBCR levels (< 300 μg/g and ≥ 300 μg/g groups). Kaplan–Meier analysis showed a significantly higher incidence of all-cause and cardiovascular mortality rates in the high UBCR group (log-rank P < 0.01). Multivariable Cox proportional hazards model adjusted for age, sex, estimated glomerular filtration rate (eGFR), urine albumin level, smoking, and comorbidities showed a significantly higher hazard ratio of 1.49 (95% confidence interval (CI) 1.10–2.03, P = 0.01) for all-cause mortality and a hazard ratio of 1.73 (95% CI 1.00–2.98, P = 0.048) for cardiovascular mortality in the high-UBCR group. The net reclassification index was significantly improved by adding a high UBCR to the conventional risk factors.

**Conclusion:**

UBCR is an independent risk factor for all-cause and cardiovascular mortality in the general population, independent of eGFR and urinary albumin levels.

**Supplementary Information:**

The online version contains supplementary material available at 10.1007/s10157-024-02592-6.

## Introduction

The global incidence of chronic kidney disease (CKD) is increasing, establishing it as a significant risk factor for progression to end-stage kidney disease and increasing the risk of cardiovascular disease (CVD) and overall mortality [1–3]. This underscores the critical need for detailed epidemiological research on the association between CKD and cardiovascular prognosis, including mortality.

Albuminuria is a well-established early indicator of kidney damage [4], and tubular damage is a crucial indicator of renal failure. The β2-microglobulin is filtered out from the glomerulus and is almost completely reabsorbed in the proximal tubules, with less than 400 ng of urinary β2-microglobulin excreted daily [5]. Since impaired reabsorption in the proximal tubules causes an increase in urinary excretion of β2-microglobulin, the level of urinary β2-microglobulin-creatinine ratio (UBCR) can be used as a marker of renal tubular damage. Our previous cohort study in the general population demonstrated that a high UBCR (≥ 300 μg/g) was independently associated with deteriorating kidney function [6]. Additionally, elevated UBCR are independent predictors of cardiovascular events and mortality in patients with heart failure, further emphasizing their prognostic significance [7, 8]. In unique conditions such as Balkan endemic nephropathy and cadmium (Cd)-contaminated areas, renal tubular damage has been implicated in mortality and cardiovascular risk [9–13].

These findings indicate that tubular damage, manifested by UBCR, could be a significant predictor of poor prognosis. However, studies examining the relationship between UBCR levels and all-cause and cardiovascular mortality rates in the general community remain lacking. To address this gap, we conducted this cohort study using UBCR as a marker of renal tubular damage in Japanese community residents.

## Materials and methods

### Study population

This study is part of an ongoing molecular epidemiology project supported by the 21^st^-century Centers of Excellence (COE) and the Global COE program. Baseline data collection was performed between 2004 and 2006, and a follow-up survey was conducted. Detailed descriptions of the study methodology, participant recruitment strategies, and demographic profile of the study population are available elsewhere [14].

**Fig. 1:**
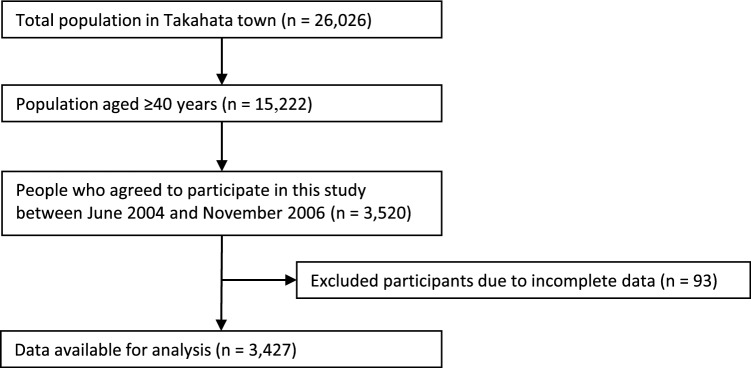
Flowchart of sample selection

In this study, all individuals aged ≥ 40 years residing in Takahata, a town in Yamagata Prefecture, were invited to undergo annual health checkups. This age group comprised 15,222 individuals (7109 men and 8113 women). Between June 2004 and November 2006, 3520 individuals (1579 men and 1941 women) agreed to participate in the study and provided written informed consent. Of the total participants, 3427 (1547 men and 1880 women) were included in the final analysis, while 93 participants with incomplete data were excluded. The follow-up period was up to December 2013 to investigate the association between urinary biomarkers and all-cause and cardiovascular mortality (Fig. 1). This study was approved by the Ethical Review Committee of the Yamagata University Faculty of Medicine (Yamagata University, April 2006, No. 1) and was conducted in accordance with the Declaration of Helsinki.

### Measurements

Participants provided information on their medical history, current medications, and clinical symptoms at the entry via a self-administered questionnaire. Blood pressure was measured in a seated position after a minimum of 5 min of rest using a mercury sphygmomanometer. Hypertension was identified if systolic blood pressure was 140 mmHg or higher, diastolic blood pressure was 90 mmHg or higher, or if the participant was taking antihypertensive drugs. Obesity was classified as having a body mass index (BMI) of 25 kg/m^2^ or higher. Diabetes was determined by blood glucose of 126 mg/dl or higher, HbA1c of 6.5% or higher, or if the participant was on medication for diabetes. Dyslipidemia was defined by LDL cholesterol of 140 mg/dL or higher, HDL cholesterol below 40 mg/dL, triglycerides of 150 mg/dL or higher, non-HDL cholesterol of 170 mg/dL or higher, or if the participant was taking medication for dyslipidemia.

The urinary albumin-creatinine ratio and UBCR were determined from a single morning spot urine sample. Urinary albumin levels were assessed using the immunoturbidimetric methods. Albuminuria was defined as an albumin concentration of 30 mg/g creatinine or higher [15]. Dipstick urinalysis results, interpreted by either a physician or a physician assistant, were categorized as (−), (±), (1+), (2+), and (3+). Proteinuria was defined as 1 + or higher. Urinary β2-microglobulin levels were assessed using the latex agglutination method, and the UBCR, adjusted for creatinine levels, was employed in the analysis. A previous study in cadmium-exposed areas revealed that high UBCR (≥ 1000 μg/g in men and UBCR ≥ 300 μg/ in women) was associated with mortality risk [12]. Our previous reports showed that the decline rate of renal function and the risk for cardiac events were significantly high in the UBCR ≥ 300 μg/g group [6–8]. Based on these findings, this study set UBCR ≥ 300 μg/g as a high UBCR cutoff value. The serum creatinine levels were measured using an enzymatic method, and the estimated glomerular filtration rate (eGFR) was obtained using a formula adapted for the Japanese population [16].

### Statistical analysis

Data were expressed as the mean ± standard deviation unless otherwise stated. Unpaired t-tests were used to assess the differences in the means, while chi-square tests were used to evaluate the differences in proportions. To examine the relationship between UBCR and all-cause and cardiovascular mortality rates, Kaplan–Meier analysis with the log-rank test and univariate and multivariate Cox proportional hazards analyses were performed to calculate the hazard ratios (HRs) and 95% confidence intervals (95% CIs). Multivariate analysis included age, gender, BMI, eGFR, alcohol consumption, smoking, hypertension, diabetes, dyslipidemia, and albuminuria as adjusting factors since these were known risk factors for all-cause and cardiovascular mortality [17–21]. Additionally, to clarify the association between UBCR and mortality further, an analysis using UBCR values classified by quartiles (Q1: < 72 μg/g, Q2: 72–111 μg/g, Q3: 112–187 μg/g, Q4: ≥ 188 μg/g) and an analysis divided by the combination of albuminuria and high UBCR ≥ 300 µg/g were performed.

To assess the predictive ability of the model including UBCR, net reclassification improvement (NRI) analysis was performed. In the NRI analysis, model 1 (baseline model) included factors used in the multivariate analysis and examined to determine whether model adding UBCR to model 1 would improve its predictive ability. A P-value of < 0.05 was considered significant. Statistical analyses were conducted using JMP Pro 16.1.0 (SAS Institute, Inc., Cary, NC) and R 4.3.2 (R Foundation for Statistical Computing, Vienna, Austria).

## Results

### Baseline characteristics

Table 1 outlines the baseline clinical characteristics of the 3427 participants. Among them, 1547 (45.1%) were men, 1114 (32.5%) were smokers, 1429 (41.7%) consumed alcohol, 1039 (30.3%) were obese, 1520 (44.4%) had hypertension, 173 (5.4%) had diabetes, and 1646 (48.0%) had dyslipidemia. Additionally, 151 participants (4.4%) exhibited proteinuria, while 512 participants (14.9%) exhibited albuminuria. A high UBCR (≥ 300 µg/g) was detected in 432 participants (12.6%). Participants with a high UBCR were typically older, demonstrated a lower estimated GFR (eGFR), were predominantly men, had hypertension, and exhibited proteinuria and albuminuria.

**Table 1:** Comparison of baseline characteristics between the UBCR < 300 µg/g group and UBCR ≥ 300 µg/g group

	Overall N = 3427	UBCR < 300 µg/g N = 2995	UBCR ≥ 300 µg/g N = 432	P-value^a^
Age (years)	62.5 ± 10.4	61.8 ± 10.3	67.4 ± 9.3	< 0.01
Male sex (%)	1547 (45.1)	1329 (44.4)	218 (50.5)	0.02
BMI (kg/m^2^)	23.5 ± 3.2	23.5 ± 3.2	23.5 ± 3.4	0.97
Systolic blood pressure (mmHg)	134.4 ± 15.8	133.4 ± 15.6	140.8 ± 15.2	< 0.01
Diastolic blood pressure (mmHg)	79.6 ± 10.1	79.2 ± 10.0	82.5 ± 10.5	< 0.01
Smoking (%)	1114 (32.5)	987 (33.0)	127 (29.4)	0.14
Alcohol consumption (%)	1429 (41.7)	1257 (42.0)	172 (39.8)	0.40
Obesity (%)	1039 (30.3)	911 (30.4)	128 (29.6)	0.74
Hypertension (%)	1520 (44.4)	1253 (41.9)	267 (61.8)	< 0.01
Diabetes (%)	173 (5.4)	148 (5.3)	25 (6.2)	0.44
Dyslipidemia (%)	1646 (48.0)	1451 (48.4)	195 (45.1)	0.22
Hypertension medication (%)	1144 (33.4)	949 (31.7)	195 (45.1)	< 0.01
HbA1c (%)	5.3 ± 0.7	5.3 ± 0.7	5.3 ± 0.7	0.50
Hemoglobin (g/dL)	13.7 ± 1.5	13.8 ± 1.5	13.6 ± 1.4	0.08
Hematocrit (%)	41.0 ± 4.1	41.1 ± 4.2	40.6 ± 4.0	0.02
Serum albumin (g/dL)	4.5 ± 0.3	4.5 ± 0.3	4.4 ± 0.3	< 0.01
Uric acid (mg/dL)	5.1 ± 1.4	5.1 ± 1.3	5.0 ± 1.4	0.10
Serum creatinine (mg/dL)	0.7 ± 0.2	0.7 ± 0.1	0.7 ± 0.5	0.09
eGFR (mL/min/1.73 m^2^)	81.6 ± 16.5	82.0 ± 16.0	78.8 ± 19.4	< 0.01
Proteinuria (%)	151 (4.4)	106 (3.5)	45 (10.4)	< 0.01
Albuminuria (%)	512 (14.9)	360 (12.0)	152 (35.2)	< 0.01
UBCR (µg/g)	111.6 (71.7–188.1)	100.1 (66.1–148.7)	468.9 (367.0–784.3)	< 0.01

Mean ± SD, n (%), median (25^th^–75^th^)

^a^ Unpaired t-test, Pearson’s chi-square test

*UBCR* urinary β2-microglobulin-creatinine ratio, *BMI* body mass index, *eGFR* estimated glomerular filtration rate

During the follow-up period (a median duration of 9.2 years), 259 participants (7.6%) died. Among them, the number of deaths from cancer, CVD, and kidney disease was 109 (42.1%), 75 (29.0%), and 4 (1.5%), respectively. Of the 66 deaths in the high UBCR group, the number of deaths from cancer, CVD, and kidney disease was 21 (31.8%), 21 (31.8%) and 3 (4.5%), respectively.

### Kaplan–Meier analysis

**Fig. 2:**
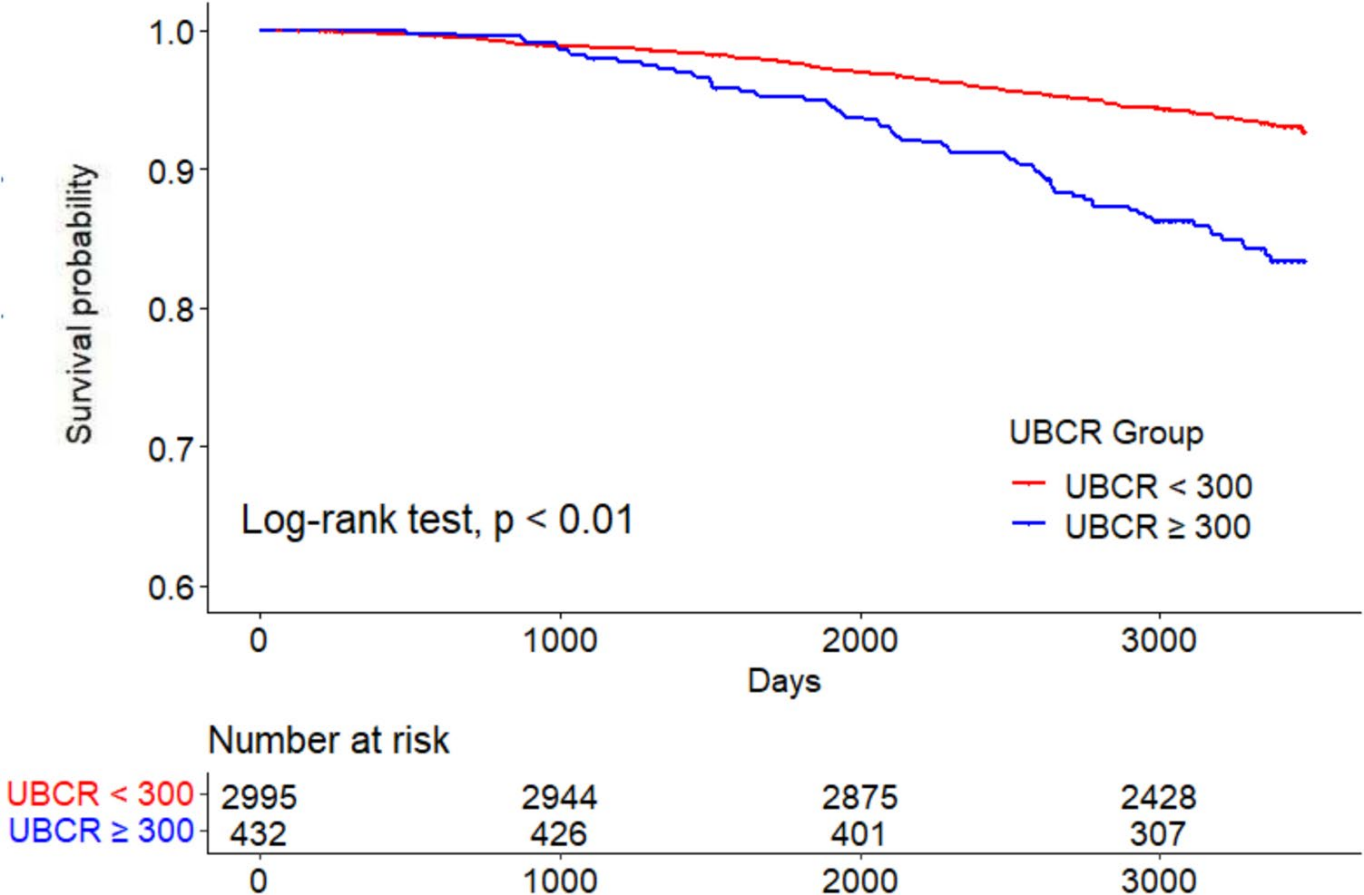
All-cause mortality in the high and low UBCR groups. *UBCR* urinary β2-microglobulin-creatinine ratio

**Fig. 3:**
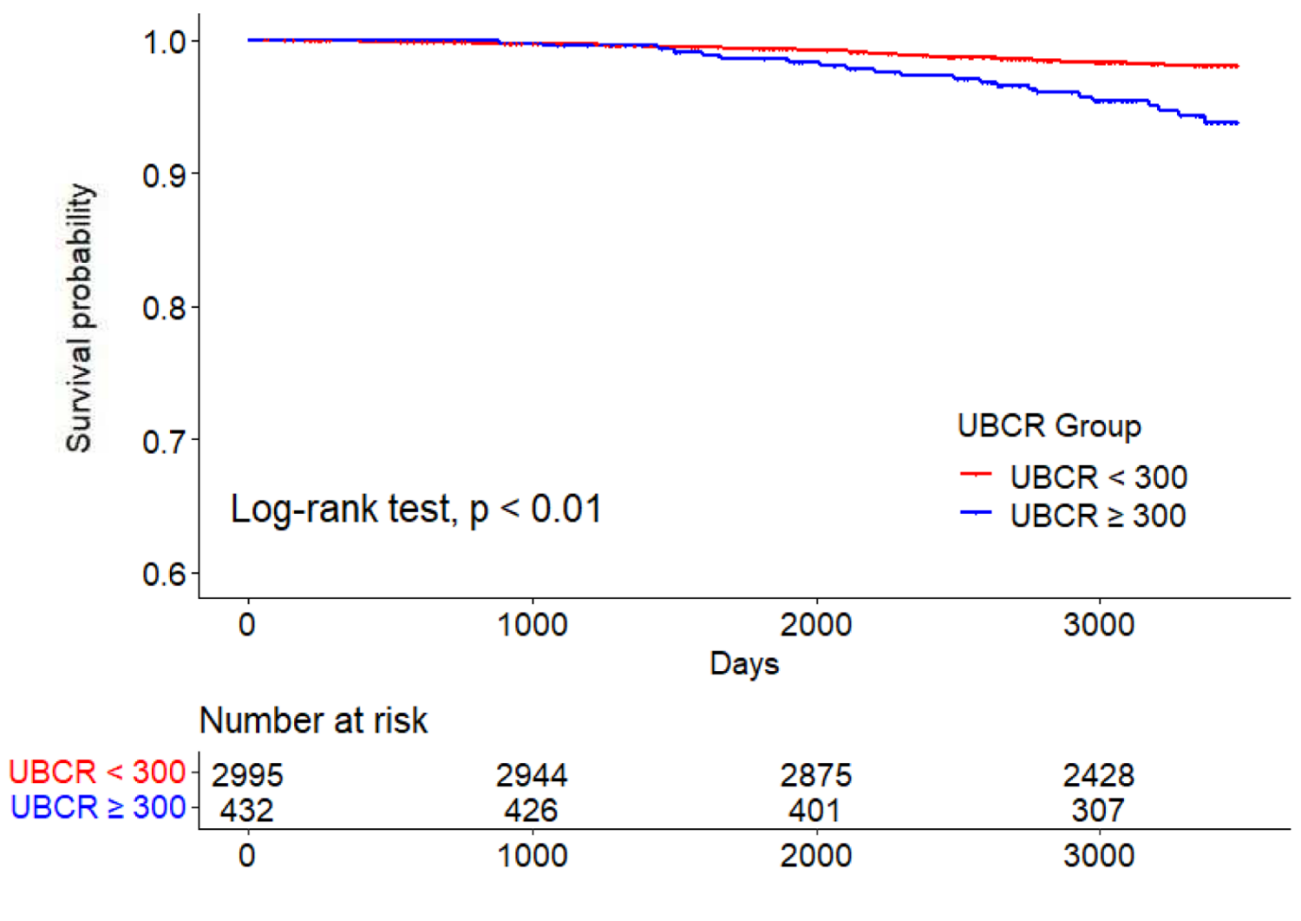
Cardiovascular mortality in the high and low UBCR groups. *UBCR* urinary β2-microglobulin-creatinine ratio

Kaplan–Meier analysis revealed that the all-cause mortality rates were significantly higher in the high UBCR group (log-rank test, P < 0.01) (Fig. 2). Similarly, the cardiovascular mortality rate was significantly higher in the high UBCR group (log-rank P < 0.01) (Fig. 3). The rates of all-cause mortality, expressed as deaths per 1000 person-years, were 2.4, 2.0, and 5.0 in all participants, low UBCR group, and high UBCR group, respectively. The rates of cardiovascular mortality, expressed as deaths per 1000 person-years, were 0.7 in all participants, 0.6 in the low UBCR group, and 1.7 in the high UBCR group, respectively.

### Cox proportional hazards model analysis

Cox proportional hazards model analysis was conducted to evaluate the impact of UBCR and known risk factors on all-cause and cardiovascular mortality rates. Univariate analysis showed that a high UBCR, along with other factors, was significantly associated with increased all-cause mortality (Table 2). When adjusting for potential confounders such as age, gender, BMI, eGFR, alcohol consumption, smoking, hypertension, diabetes, dyslipidemia, and albuminuria, the multivariable analysis revealed that a high UBCR was independently associated with all-cause mortality (HR: 1.49, 95% CI 1.10–2.03, P = 0.01) (Table 2). Further, the multivariable analyses showed an independent association between high UBCR and cardiovascular mortality (HR: 1.73, 95% CI 1.00–2.98, P = 0.048) (Table 3). Similar independent associations were observed in the multivariable analyses, including proteinuria instead of albuminuria for both all-cause (HR: 1.47, 95% CI 1.09–2.00, P = 0.01) and cardiovascular mortality rates (HR: 1.82, 95% CI 1.16–3.12, P = 0.03).

**Table 2:** Univariable and multivariable Cox proportional hazard analyses of all-cause mortality

	Univariable analyses		P-value	Multivariable analyses		P-value
	HR	95% CI		HR	95% CI	
Age (per year increase)	1.12	1.10–1.14	< 0.01	1.10	1.08–1.12	< 0.01
Male sex	2.91	2.24–3.80	< 0.01	2.36	1.64–3.41	< 0.01
BMI (per kg/m^2^ increase)	0.94	0.90–0.98	< 0.01	0.95	0.91–0.99	0.02
eGFR (per mL/min/1.73 m^2^ increase)	0.97	0.97–0.98	< 0.01	0.99	0.98–1.00	0.03
Alcohol consumption	1.26	0.98–1.60	0.07	0.97	0.71–1.32	0.83
Smoking	1.75	1.37–2.23	< 0.01	1.42	1.04–1.94	0.03
Hypertension	1.43	1.12–1.83	< 0.01	1.06	0.81–1.39	0.66
Diabetes	2.25	1.49–3.38	< 0.01	1.65	1.08–2.52	0.02
Dyslipidemia	0.73	0.57–0.93	0.01	0.95	0.72–1.24	0.71
Albuminuria	2.18	1.66–2.87	< 0.01	1.22	0.89–1.68	0.21
UBCR ≥ 300 (µg/g)	2.48	1.88–3.28	< 0.01	1.49	1.10–2.03	0.01

*HR* hazard ratio, CI confidence interval, *BMI* body mass index, *eGFR* estimated glomerular filtration rate, *UBCR* urinary β2-microglobulin-creatinine ratio

**Table 3:** Univariable and multivariable Cox proportional hazard analyses of cardiovascular mortality

	Univariate analyses		P-value	Multivariate analyses		P-value
	HR	95% CI		HR	95% CI	
Age (per year increase)	1.14	1.10–1.17	< 0.01	1.12	1.08–1.16	< 0.01
Male sex	3.53	2.11–5.89	< 0.01	1.74	0.84–3.58	0.14
BMI (per kg/m^2^ increase)	0.99	0.92–1.06	0.83	1.00	0.93–1.09	0.91
eGFR (per mL/min/1.73 m^2^ increase)	0.97	0.96–0.98	< 0.01	0.99	0.98–1.00	0.16
Alcohol consumption	1.73	1.10–2.72	0.02	1.24	0.69–2.24	0.46
Smoking	2.47	1.57–3.88	< 0.01	2.09	1.15–3.79	0.02
Hypertension	1.81	1.14–2.87	0.01	1.24	0.75–2.05	0.39
Diabetes	3.07	1.57–6.00	< 0.01	2.06	1.02–4.18	0.04
Dyslipidemia	0.58	0.36–0.93	0.02	0.76	0.46–1.26	0.29
Albuminuria	2.37	1.09–5.17	0.03	1.41	0.81–2.44	0.22
UBCR ≥ 300 (µg/g)	2.95	1.83–4.77	< 0.01	1.73	1.00–2.98	0.048

*HR* hazard ratio, *CI* confidence interval, *BMI* body mass index, *eGFR* estimated glomerular filtration rate, *UBCR* urinary β2-microglobulin-creatinine ratio

Additional multivariate Cox proportional hazards analysis was performed by classifying UBCRs into quartiles (Q1: < 72 μg/g, Q2: 72–111 μg/g, Q3: 112–187 μg/g, Q4: ≥ 188 μg/g). It revealed that the Q4 group showed high risk for all-cause mortality (HR 1.46, 95% CI 1.00–2.14, P = 0.048) and for cardiovascular mortality (HR 1.51, 95% CI 0.76–3.01, P = 0.24) as compared with Q1 (Supplementary Table 1). Further, the analysis divided by the combination of albuminuria and high UBCR was performed. The number of subjects were 2635 (76.9%) with albuminuria/high UBCR (−)/(−), 360 (10.5%) with (+)/(−), 280 (8.2%) with (−)/(+), and 152 (4.4%) with (+)/(+), respectively. Multivariate Cox proportional hazards analysis showed that a significant difference was observed only between the (−)/(−) and (+)/( +) groups in all-cause and cardiovascular mortality (Supplementary Table 2).

For mediation analysis, additional variables were incorporated into the baseline multivariate Cox proportional hazards model. Factors correlated with UBCR, including hemoglobin (Hb), fibrinogen, high-sensitivity C-reactive protein (CRP), serum albumin, uric acid, urinary sodium creatinine ratio, urinary calcium creatinine ratio, and urinary phosphorus creatinine ratio, were incorporated into the model. Despite the inclusion of these variables, the independence of UBCR remained unchanged (Supplementary Table 3).

### Predictability of the model, including UBCR

We used the NRI to examine the added value of a high UBCR (≥ 300 µg/g) in predicting mortality. The model adding high UBCR to model 1 including age, gender, BMI, eGFR, alcohol consumption, smoking, hypertension, diabetes, dyslipidemia, and albuminuria showed significantly higher predictive accuracy than baseline model 1, with NRI values of 0.184 (95% CI 0.066–0.301, P < 0.01) for all-cause mortality and 0.304 (95% CI 0.083–0.526, P < 0.01) for cardiovascular death (Table 4).

**Table 4:** Predictability performance of the all-cause and cardiovascular mortality prediction model with or without UBCR

	All-cause mortality			Cardiovascular mortality		
	NRI		P-value	NRI		P-value
	index	95% CI		index	95% CI	
Model 1 (baseline model)						
Model 2 (Model 1 + UBCR)	0.184	0.066–0.301	< 0.01	0.304	0.083–0.526	< 0.01

Model 1 (baseline model) incorporates age, male sex, BMI, eGFR, Alcohol consumption, smoking, hypertension, diabetes, dyslipidemia, and albuminuria

*UBCR* urinary β2-microglobulin-creatinine ratio, *NRI* net reclassification improvement, *CI* confidence interval, *eGFR* estimated glomerular filtration rate

## Discussion

This study showed that high UBCR was significantly associated with an increased risk of all-cause and cardiovascular mortality in the local Japanese residents. This association remained significant in the multivariate analyses that included known risk factors such as eGFR and urinary albumin. Furthermore, incorporating the UBCR into prediction models improved the accuracy of predicting these mortalities. These findings suggest that high UBCR is an independent risk factor for all-cause and cardiovascular mortality in the general population.

The prevalence rate of high UBCR in this study was 12.6%, which is lower than the rate observed in cadmium-polluted areas in Japan (33.7%) [10] and in patients with heart failure who developed chronic kidney disease (60%), but similar to the rate in residents of Zimbabwe (8% in normotensives and 11% in hypertensives) [22]. Although data on the prevalence of high UBCR among healthy Japanese residents are lacking, our findings may be applicable to the general Japanese population.

Our observation of increased mortality risk at high UBCR levels aligns with that of previous studies conducted in special settings, such as patients with heart failure and residents of Cd-polluted areas [8, 9, 12]. Our study is notable owing to its large cohort of community-based individuals. This suggests that even minor tubular damage could be a significant risk, underscoring the need to pay more attention to renal tubular damage in the general population.

Previous studies have shown that impaired renal function and albuminuria are risk factors for mortality [1]. Our findings further indicate that renal tubular damage is an independent predictor of all-cause and cardiovascular mortality, even when eGFR and albuminuria were considered. Of interest, albuminuria, a marker of glomerular damage and systemic microvascular damage [23] and a well-known risk for mortality, was not significantly associated with all-cause and cardiovascular mortality in this study population. Although the reason for this finding is not clear, one possible explanation is that there was a relatively small number of cardiovascular deaths that were strongly associated with albuminuria. Another possible explanation is that high albuminuria directly causes renal tubular damage [24]; therefore, some part of high UBCR might be due to the advanced renal damage caused by albuminuria. The finding from an additional analysis that high UBCR increases risk only in the case of albuminuria ( +) suggests that high UBCR might have an additive effect in combination with albuminuria. Further investigation on how UBCR value can be interpreted and used as a risk marker in the general population is necessary.

High UBCR was reported to be a risk factor for CKD and CVD [6, 7]. However, more than half of the total deaths in the high UBCR group were not directly related to CKD or CVD in this study. This indicates that UBCR levels are related to not only CKD/CVD-related deaths but a variety of causes of death. In additional analyses in which Hb, fibrinogen, high-sensitivity CRP, serum albumin, uric acid, urinary sodium creatinine ratio, urinary calcium creatinine ratio, and urinary phosphorus creatinine ratio were added to the Cox proportional hazards model, high UBCR remained an independent risk factor for all-cause and cardiovascular mortality. This finding suggests that anemia, undernutrition, inflammation, hyperuricemia, and urinary electrolytes (sodium, calcium, and phosphorus) are unlikely to mediate as single factors in the association between UBCR and mortality.

As the strength, the present study included a healthy general population and utilized a relatively unbiased, large, and long-term follow-up cohort design. UBCR could be an additional biomarker for identifying individuals at high risk of mortality in the general population. However, this study has several limitations. First, this study was conducted on a single ethnic group of Japanese people in a specific region, potentially introducing selection bias. Therefore, caution is necessary when generalizing the results of this study to larger populations. Second, the analysis was based on baseline data and did not account for changes in urinary findings over time or individual physiological variations. Third, owing to the nature of the epidemiological study, the detailed mechanisms by which tubular damage is a risk factor for death could not be identified.

In conclusion, renal tubular damage was a predictor of all-cause and cardiovascular mortality independent of eGFR and urinary albumin levels in the general Japanese population. To clarify the mechanisms of renal tubular damage and its pathological roles involved in the risk of mortality, further investigation is warranted.

## Supplementary Information

Below is the link to the electronic supplementary material.

Supplementary file1 (DOCX 40 KB)
