# Supplementary material for: Renal tubular damage as an independent risk factor for all-cause and cardiovascular mortality in a community-based population: the Takahata study
Source: Clin Exp Nephrol. 2024 Nov 14;29(4):444–51. doi: 10.1007/s10157-024-02592-6 (PMC11937237; doi:10.1007/s10157-024-02592-6)
Supplement: Supplementary file 1 — Supplementary file1 (DOCX 40 KB) [file 10157_2024_2592_MOESM1_ESM.docx]

**Supplementary Table 1. Univariable and multivariable Cox proportional hazards analyses of all-cause and cardiovascular mortality by UBCR quartiles.**

|  | | Univariate analyses | | P-value | Multivariate analyses* | | P-value |
| --- | --- | --- | --- | --- | --- | --- | --- |
|  |  | HR | 95% CI |  | HR | 95% CI |  |
| All-cause mortality | |  |  |  |  |  |  |
| UBCR | Q1 (<72 μg/g) | Reference |  |  | Reference |  |  |
|  | Q2 (72-111 μg/g) | 0.94 | 0.63-1.42 | 0.77 | 1.16 | 0.77-1.79 | 0.49 |
|  | Q3 (112-187 μg/g) | 1.33 | 0.91-1.94 | 0.13 | 1.22 | 0.81-1.85 | 0.34 |
|  | Q4 (≥188 μg/g) | 2.31 | 1.64-3.25 | <0.01 | 1.46 | 1.00-2.14 | 0.048 |
| Cardiovascular mortality | |  |  |  |  |  |  |
| UBCR | Q1 (<72 μg/g) | Reference |  |  | Reference |  |  |
|  | Q2 (72-111 μg/g) | 0.82 | 0.38-1.75 | 0.61 | 1.21 | 0.54-2.69 | 0.65 |
|  | Q3 (112-187 μg/g) | 1.03 | 0.50-2.11 | 0.93 | 1.08 | 0.50-2.36 | 0.84 |
|  | Q4 (≥188 μg/g) | 2.32 | 1.26-4.27 | <0.01 | 1.51 | 0.76-3.01 | 0.24 |
| HR: hazard Ratio, CI: confidence interval, BMI: body mass index, eGFR: estimated glomerular filtration rate, UBCR: urinary β2-microglobulin-creatinine ratio | | | | | | | |
| * The analysis was adjusted for age, gender, BMI, eGFR, alcohol consumption, smoking, hypertension, diabetes, dyslipidemia, and albuminuria. | | | | | | | |

**Supplementary Table 2. Univariate and multivariate Cox proportional hazards analysis of all-cause and cardiovascular mortality by combining albuminuria and high UBCR (≥300 µg/g).**

|  | Univariate analyses | | P-value | Multivariate analyses* | | P-value |
| --- | --- | --- | --- | --- | --- | --- |
|  | HR | 95% CI |  | HR | 95% CI |  |
| All-cause mortality |  |  |  |  |  |  |
| Albuminuria(-) / high UBCR(-) | Reference |  |  | Reference |  |  |
| (+) / (-) | 1.76 | 1.23-2.52 | <0.01 | 1.07 | 0.72-1.60 | 0.74 |
| (-) / (+) | 2.04 | 1.40-2.97 | <0.01 | 1.31 | 0.88-1.95 | 0.19 |
| (+) / (+) | 4.02 | 2.76-5.85 | <0.01 | 2.01 | 1.33-3.03 | <0.01 |
| Cardiovascular mortality |  |  |  |  |  |  |
| Albuminuria(-) / high UBCR(-) | Reference |  |  | Reference |  |  |
| (+) / (-) | 2.18 | 1.15-4.15 | 0.02 | 1.07 | 0.53-2.24 | 0.81 |
| (-) / (+) | 2.12 | 1.03-4.35 | 0.04 | 1.29 | 0.88-1.95 | 0.52 |
| (+) / (+) | 6.04 | 3.23-11.27 | <0.01 | 2.78 | 1.41-5.46 | <0.01 |
| HR: hazard Ratio, CI: confidence interval, BMI: body mass index, eGFR: estimated glomerular filtration rate, UBCR: urinary β2-microglobulin-creatinine ratio | | | | | | |
| *The analysis was adjusted for age, gender, BMI, eGFR, alcohol consumption, smoking, hypertension, diabetes, and dyslipidemia. | | | | | | |

**Supplementary Table 3. Changes in the hazard ratios for high UBCR in the multivariate Cox proportional hazards models of all-cause mortality (with additional mediating factors).**

|  | **All-cause mortality** | | **P-value** | **Cardiovascular mortality** | | **P-value** |
| --- | --- | --- | --- | --- | --- | --- |
|  | **HR** | **95% CI** |  | **HR** | **95% CI** |  |
| Baseline model | 1.49 | 1.10–2.03 | 0.01 | 1.73 | 1.06–2.98 | 0.048 |
| + Anemia | 1.47 | 1.08–2.01 | 0.01 | 1.67 | 0.97–2.88 | 0.07 |
| + Fibrinogen | 1.53 | 1.13–2.09 | <0.01 | 1.77 | 1.03–3.05 | 0.04 |
| + High sensitive CRP | 2.00 | 1.21–3.32 | <0.01 | 2.33 | 0.77–7.07 | 0.13 |
| + Serum albumin | 1.42 | 1.04–1.93 | 0.03 | 1.61 | 0.93–2.78 | 0.09 |
| + Urine sodium creatinine ratio | 1.72 | 1.24–2.39 | <0.01 | 1.95 | 1.09–3.49 | 0.03 |
| + Urine calcium creatinine ratio | 1.84 | 1.20–2.83 | <0.01 | 1.75 | 0.72–4.23 | 0.21 |
| + Urine phosphorus creatinine ratio | 1.82 | 1.18–2.79 | <0.01 | 1.68 | 0.70–4.05 | 0.25 |
| + GNRI-BMI+ | 1.43 | 1.05–1.95 | 0.02 | 1.67 | 0.97–2.89 | 0.07 |
| HR: hazard ratio, CI: confidence interval, BMI: body mass index, eGFR: estimated glomerular filtration rate, UBCR: urinary β2-microglobulin-creatinine ratio  Baseline model incorporates age, male sex, BMI, eGFR, consumption of alcohol, smoking, hypertension, diabetes, dyslipidemia, albuminuria, and UBCR ≥300 (µg/g).  Anemia; men Hb ≤13 g/dl, women Hb ≤12 g/dl  GNRI-BMI (Geriatric Nutritional Risk Index): 14.89×Alb (g/dl) + 41.7×BMI/22 | | | | | | |
